# Could Flow Cytometry Provide New Prognostic Markers in Colorectal Cancer?

**DOI:** 10.3390/jcm13164753

**Published:** 2024-08-13

**Authors:** Vaia Georvasili, Georgios Markopoulos, Evangeli Lampri, Georgios Lianos, George Vartholomatos, Michail Mitsis, Christina Bali

**Affiliations:** 1 Department of Surgery, University Hospital of Ioannina, 45500 Ioannina, Greece; giouli.geo77@gmail.com (V.G.); glianos@uoi.gr (G.L.); mmitsis@uoi.gr (M.M.); 2 Unit of Molecular Biology, University Hospital of Ioannina, 45500 Ioannina, Greece; geomarkop@gmail.com (G.M.); gvarthol@gmail.com (G.V.); 3 Department of Pathology, University Hospital of Ioannina, 45500 Ioannina, Greece; elampri@uoi.gr

**Keywords:** flow cytometry, DNA index, aneuploidy, tumor index, CD26, colorectal cancer

## Abstract

**Background/Objectives**: Colorectal cancer (CRC) is still accompanied by significant mortality, which poses the necessity of novel markers to predict treatment success and patient survival. This study aims to evaluate the prognostic and survival impact of flowytometry (FC) in CRC patients. **Methods**: In this prospective study, 106 surgically resectable CRC patients were included. Tissue specimens from tumor and normal mucosa were collected and analyzed by FC. DNA and tumor index were calculated. In a subgroup of 46 patients, the CD26 expression on tumor cells was estimated. These parameters were compared with patients’ tumor characteristics as stage, histology data, responsiveness to treatment, metastasis/recurrence, and, finally, patients’ survival to identify possible new biomarkers. **Results**: The overall survival and the disease-specific survival in our study group was 76% and 72%, respectively, during the 7-year follow up period. Diploid tumors had better median survival than the aneuploid ones. The DNA index had significant correlation to the tumor index and response to neoadjuvant treatment. Similarly, the tumor index was also significantly related to the response to neoadjuvant treatment. Patients with a higher tumor index had worst survival rates. Surprisingly, CD26 levels were not associated with any of the parameters examined and were negatively related to tumor stage and differentiation. **Conclusions**: FC is a rapid and reliable method of cell analysis. In CRC, it has been used for prognostic and diagnostic purposes. In this study, we have shown that DNA and tumor index could become predictive biomarkers of tumor response to neoadjuvant treatment and survival of resectable CRC patients.

## 1. Introduction

Colorectal cancer (CRC) is currently the third most frequent human malignancy [1]. Despite advances in surgical techniques and adjuvant/neoadjuvant therapies, CRC remains a lethal disease, with almost one million deaths in 2020 [1]. This highlights the necessity of tailoring the provided treatment to every patient, mostly depending on the specific tumor characteristics and biology.

Currently, CRC features like tumor stage and grade, lymph node ratio, perineural and lymphovascular invasion, MSI status, tumor budding, circumferential resection margin invasion, tumor regression score, and mutation in KRAS/NRAS and BRAF oncogenes are utilized in treatment strategy and prediction of disease prognosis [2,3]. New prognostic biomarkers in CRC are under investigation, but their clinical utility has not been established yet [4]. In the past, several studies have highlighted the role of tumor aneuploidy as an independent prognostic marker in CRC [5,6]. Aneuploidy is an abnormality in the number of chromosomes due to loss or duplication, and the cell has a number of chromosomes other than 46, which is the number of chromosomes of normal diploid cells.

Flow cytometry (FC) is a technique based on laser technology that analyzes the properties of a particle or a cell. FC has been used for the evaluation of cellular phenotype and analysis of parameters such as cell proliferation and death, and is the most widely used method to detect aneuploidy [6]. FC quantifies the DNA ploidy by the estimation of the DNA index; estimates the proliferative potential of a cell, defined as the tumor index (TI); and discriminates cells depending on the expression of different cell surface proteins, like CD15, CD44, CD133, and CD26. These proteins and their role as biomarkers in CRC have been studied in past years [7,8,9]. Particularly, CD26 is considered a significant biomarker of cancer stem cells in CRC, which can induce tumor metastasis and provide chemotherapy resistance to cancer cells. High levels of CD26 are considered a predictor of worse outcome in CRC patients [9,10].

In our previous study, intraoperative FC showed a 91% accuracy in the detection of tumor cells in fresh tissue specimens of CRC based on TI values. TI was significantly higher (*p* < 0.05) in all cancer cases, irrespective of the tumor stage [8].

Following the completion of the follow-up, the aim of this study is to evaluate the prognostic and survival impact of DNA ploidy, TI, and expression of CD26 in CRC patients.

## 2. Materials and Methods

One hundred and six patients with biopsy-proven CRC adenocarcinoma underwent elective colectomy between 1/2017 and 8/2021 and were prospectively included in this study group. Two specialized colorectal surgeons performed the operations according to the current treatment guidelines. Tissue samples from tumor and normal colon mucosa were collected, analyzed, and compared by FC intraoperatively using the Ioannina Protocol, as we fully described in our previous publication [8]. All patients’ data, tumor characteristics (tumor location, stage, and differentiation; perineural/lymphovascular invasion; tumor budding; lymph node ratio [LNR]; and tumor regression grade [TRG]) and applied treatment were recorded. In patients that had received neoadjuvant chemo/radiation (nCRT) therapy, TRG was estimated in colon specimens by pathologists based on the four categories of AJCC/CAP TRG: grade 0 (complete response), no remaining viable cancer cells; grade 1 (near complete response), only small cluster or single cancer cells remaining; grade 2 (partial response), residual cancer remaining, but with predominant fibrosis; grade 3 (poor or no response), extensive residual cancer [11]. FC parameter analysis of the DNA index, TI, and G0/G1 cell fraction were used in the detection of cancer cells. The DNA index quantifies cancer cell DNA by comparing the fluorescence intensity of cancer cells in relation to a normal diploid cell. A DNA index value of 1.0 suggests a typical diploid DNA of normal cells, and all other values (<1 or >1) suggest the presence of aneuploidy, which constitutes a common characteristic of cancer cells [6,12]. A deviation greater than 5% in DNA index has been used as the cut-off value to define aneuploidy, as previously described (<0.95 for hypoploidy and >1.05 for hyperploidy) [13]. The tumor index corresponds to the proportion of the proliferating cancer cells in a tissue sample. It is calculated based on the percentage of cells in the S and G2/M phases of the cell cycle.

Additionally, in 45 consecutive patients, the expression levels of CD26 were measured by flow cytometry using a CD26-specific antibody (clone L272, BD Biosciences, NJ, USA) using standard methodologies [14]. Briefly, cells were incubated with CD26 antibody according to the manufacturer’s instructions and incubated for 10 min at room temperature (18–25 °C) while protected from light. Samples were processed immediately for analysis. The data were analyzed using the CellQuest v.3.1 software (Becton Dickinson, Franklin Lakes, NJ, USA).

At the end of the follow-up period in 1/2024, the overall survival, cancer-related death, disease-free survival (DFS), and local and distant metastasis rate were recorded. We evaluated the impact of these FC parameters on patient prognosis and survival, and their possible utilization as CRC biomarkers.

### Statistical Analysis

The Mann–Whitney U test was employed for comparison purposes, and Kaplan–Meier analysis was utilized to assess overall survival and disease-free survival. Bivariate correlations were analyzed using both Pearson and Spearman methods. Continuous data were represented by the mean and standard deviation. A probability value of below 0.05 was set as the threshold for statistical significance. Statistical analyses were conducted using SPSS V.23 software (IBM, Armonk, NY, USA), and the results were visualized with GraphPad Prism V 8.4.2 (GraphPad Software, LLC, Boston, MA, USA).

## 3. Results

The study group consisted of 106 consecutive CRC patients. Patients’ data are summarized in Table 1. Most patients were males. The location of the tumors was almost equally distributed in the right colon, left colon, and rectum. Eighteen patients received neoadjuvant chemo/radiation, of which 13 patients had rectal/rectosigmoid cancer. All patients had R0 resection.

The median follow-up period was 35 months (range: 4–82 months). During this period, 22 patients (20.7%) developed recurrence or/and metastasis of their neoplasm, with most of them being stage III and IV or having a high TRG. The overall survival (OS) and disease-free survival (DFS) was 76% and 72%, respectively. As expected, OS was negatively related to stage progression, although it did not reach statistical significance.

**Figure 1:**
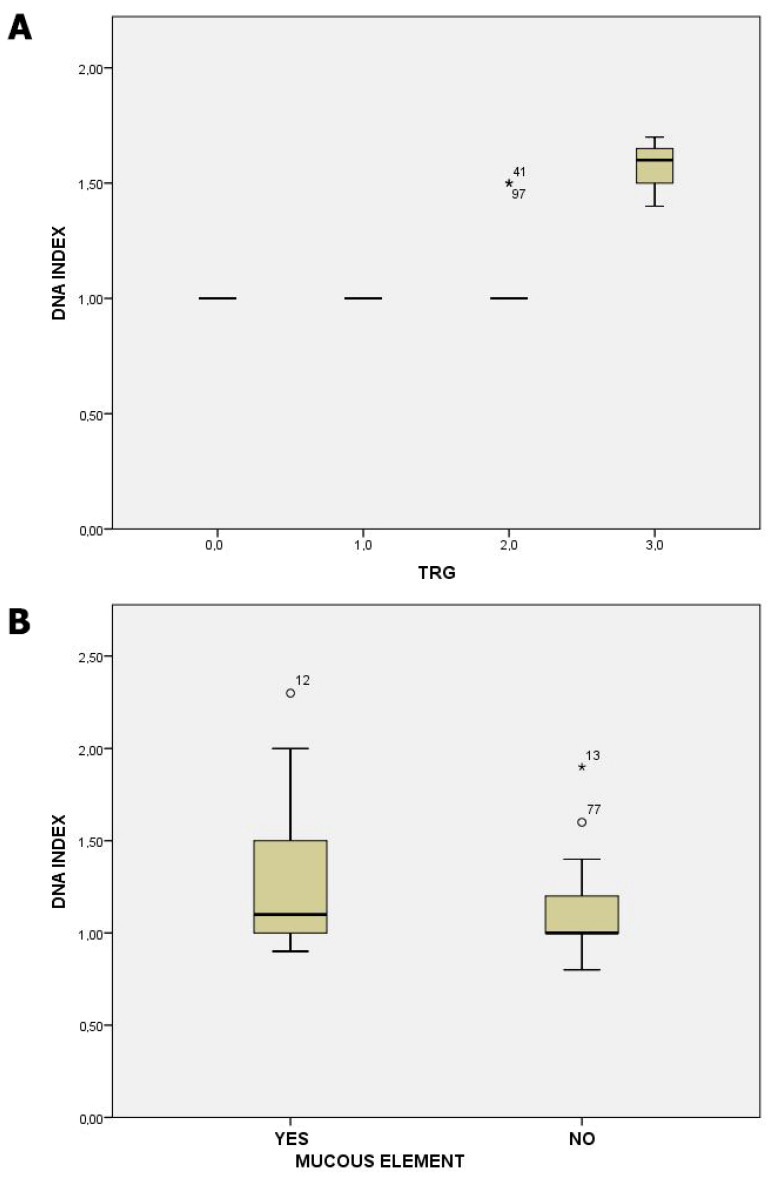
Panel (**A**): graph depicting the relationship between DNA index and tumor regression grade (TRG). The plot illustrates the association between the cellular DNA content and the response of tumors to treatment, as determined using the TRG system. Panel (**B**): graph displaying the correlation between DNA index and mucous element presence within all the tumors in the study group. This plot reveals the association between DNA content variability and the extent of mucous production by tumor cells, highlighting potential prognostic implications. Points labeled with sample numbers indicate cases with values more than two standard deviations from the mean.

Regarding FC analysis, most CRC tumors were aneuploid (55%). The DNA index had a statistically significant correlation only to the tumor index and TRG (*p*: 0.01) and was negatively correlated to mucinous colorectal tumors (*p*: 0.05) [Figure 1].

**Figure 2:**
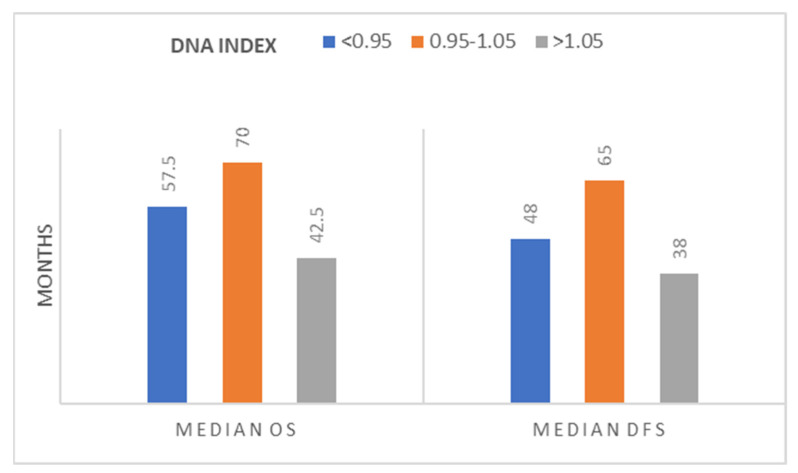
Median overall and disease-free survival in association with the tumor’s DNA index. Groups <0.95, 0.95–1.05, and >1.05 correspond to hypoploid, diploid (with an acceptable standard deviation of 5%), and hyperploid, as measured by intraoperative flow cytometry.

We did not confirm any association between DNA index and any of the parameters examined (sex, tumor location, stage, differentiation, perineural/lymphovascular invasion, tumor budding, lymph node ratio [LNR], recurrence). The median OS and DFS was higher in diploid tumors in comparison to aneuploid ones, but the difference was not statistically significant (Figure 2).

**Figure 3:**
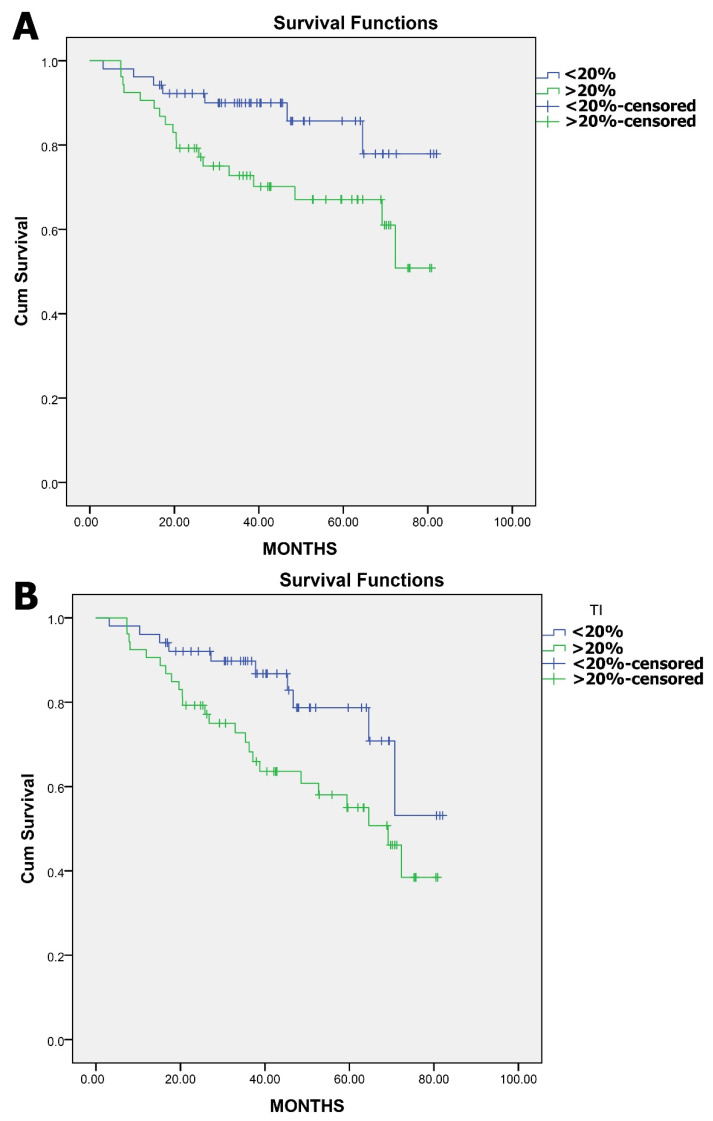
Panel (**A**) Kaplan–Meier survival curve illustrating overall survival in patients with colorectal tumors, categorized by low and high tumor index. This graph compares the overall survival probabilities over time between the two groups, highlighting differences in patient outcomes based on tumor index categorization. Panel (**B**): Kaplan–Meier survival curve depicting disease-free survival (DFS) in patients with low- and high-tumor-index colorectal tumors. This curve demonstrates the duration of survival without signs of disease recurrence or progression, contrasting the prognostic impacts of varying tumor index levels.

On the other hand, the TI showed a trend of increasing with the advancement of tumor stage, but this was not proven to be statistically significant. Based on our previous work [8], the median TI of our population was calculated to be 20%. We analyzed two groups with equal populations of 53 patients with low and high tumor indexes based on the median. The TI was significantly related to the patient survival based on the cut-off value of the median TI of 20%. As shown in the survival curves, higher TI values led to worst OS and DFS [Figure 3].

**Figure 4:**
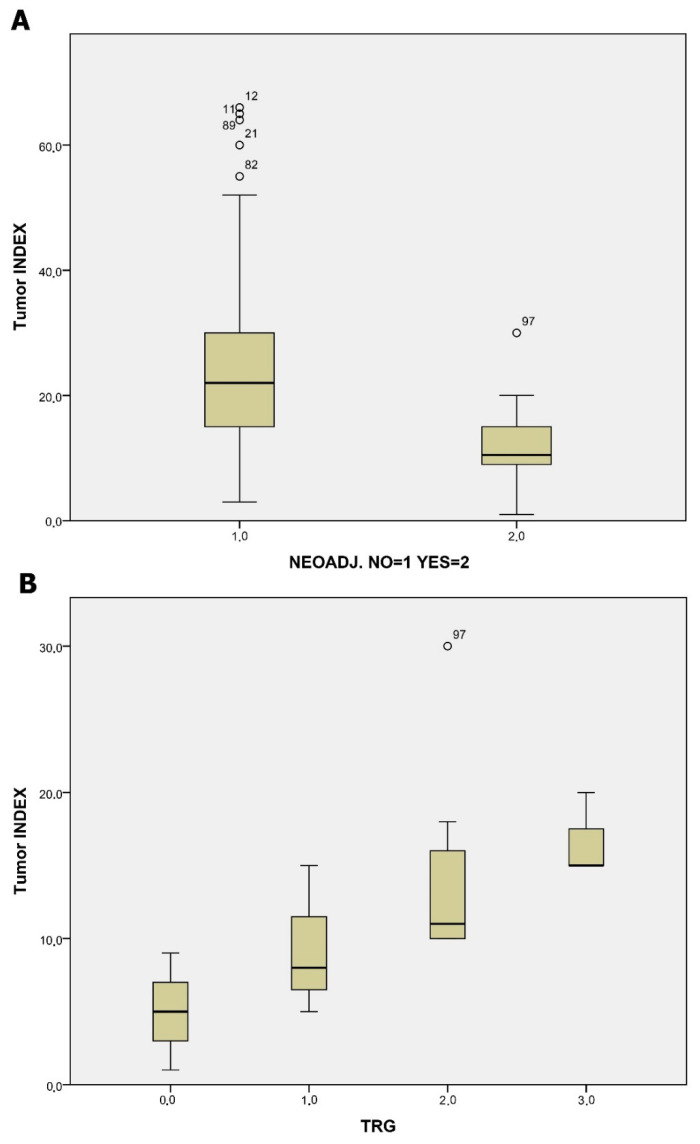
Panel (**A**): box plot presenting the distribution of tumor index (TI) values among patients undergoing neoadjuvant therapy for colorectal cancer. This graph highlights the statistical correlation between TI levels and the administration of neoadjuvant treatment (*p* < 0.01), showcasing how TI can vary with treatment application. Panel (**B**): box plot showing the relationship between tumor index (TI) and tumor regression grade (TRG) following neoadjuvant treatment. Lower TI values are associated with better TRG outcomes, indicating a more effective response to treatment. The plot confirms the statistical significance of this relationship (*p* < 0.01). Points labeled with sample numbers indicate cases with values more than two standard deviations from the mean.

Another significant association was found between TI and the administration (*p*: 0.05) and response (TRG) (*p*: 0.01) to neoadjuvant treatment. Patients who had received neoadjuvant treatment and those with better response had lower TI values, as shown in Figure 4.

**Figure 5:**
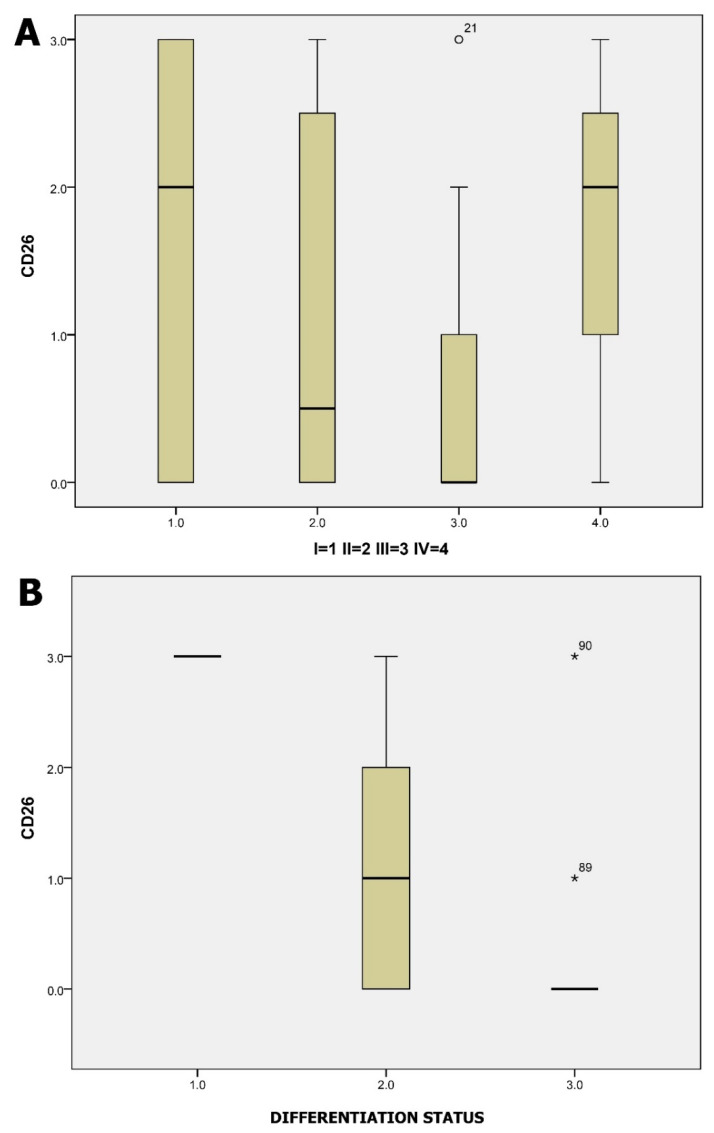
In these histograms, the levels of CD26 expression by tumor cells are shown, categorized into four groups: absent (0), low (1), moderate (2), and high (3). Panel (**A**) displays the distribution of CD26 expression across different CRC stages (1 = stage I, 2 = stage II, 3 = stage III, 4 = stage IV), while panel (**B**) illustrates the association between CD26 expression levels and tumor differentiation grades (numbers in axis represent 1 = well differentiated, 2 = moderate differentiation, 3 = poor differentiation). Analysis was performed using intraoperative flow cytometry (iFC). Points labeled with sample numbers indicate cases with values more than two standard deviations from the mean.

The characteristics of patients analyzed for CD26 expression are presented in Table S1. Regarding the expression of CD26 in tumor cells, we found no statistically significant association with any of the parameters examined, except for tumor stage and differentiation. The distribution of CD26 levels in different CRC stages and differentiation grades is shown in Figure 5. Surprisingly, in our patients, there was a negative statistical correlation to CRC stage and differentiation (*p*: 0.05).

## 4. Discussion

Colorectal cancer (CRC) is a complex disease characterized by a series of genetic and epigenetic alterations that drive tumorigenesis. The pathogenesis of CRC is generally understood to follow a progression from benign adenomatous polyps to malignant carcinoma, a process that involves multiple genetic mutations and the activation of oncogenic pathways [15]. In this study, we tried to determine the possible implication of FC in the prediction of the prognosis and survival of CRC patients. Tumorigenesis in CRC is driven by different pathways. Chromosomal instability (CIN) is the most common pathway (80%) and the main cause of genomic instability [16,17].

Calculation of the DNA index by FC is currently utilized to detect aneuploidy. Aneuploidy, a result of genomic instability, a hallmark of cancer, is the presence of an abnormal number of chromosomes within a cell [18]. In CRC, aneuploidy often results from defects in the mitotic checkpoint, leading to chromosomal missegregation [19]. This genomic instability promotes tumor heterogeneity, enabling the selection of more aggressive cancer cell clones [20]. Aneuploidy has been associated with increased tumor cell proliferation, evasion of apoptosis, and enhanced metastatic potential [6,20]. Our study observed a 51% incidence of aneuploidy in CRC patients, aligning with existing literature [21]. Despite its prevalence, the prognostic significance of aneuploidy in CRC remains controversial and currently is not widely accepted as a prognostic biomarker in CRC [18,19,20,22,23].

Laubert et al., in their metanalysis, showed that there is a trend towards aneuploidy in late CRC stages, but only in half of analyzed studies did it have a significant prognostic impact for overall, disease-specific, and recurrence-free survival [21]. In our statistical analysis, we found that patients with diploid tumors had better OS and DFS, which is in agreement with the relevant literature, although this did not reach statistical significance [24,25]. This underscores the complexity of aneuploidy as a biomarker and highlights the need for further research. Additionally, we showed a statistically significant association of DNA index to TRG and mucinous tumors. Hyperploid tumors were related to unresponsiveness to neoadjuvant CRT and lacking in mucinous elements in comparison to diploid ones. All the other tumor parameters examined, including the stage, did not reach any significance in relation to DNA index.

The tumor index (TI), a measure of tumor cell proliferation and aggressiveness, serves as a quantification method of crucial hallmarks of cancer [18]. This index reflects the rate at which cancer cells divide and expand, providing insights into the biological behavior of the tumor. A high TI is often indicative of a more aggressive cancer phenotype, characterized by rapid growth and a higher potential for invasion and metastasis [8,16,26]. By quantifying the proliferative capacity of tumor cells, the TI offers valuable prognostic information that might guide treatment decisions and help tailor therapeutic approaches to individual patient needs. In our study, tumor index was significantly related to OS, DFS, and the administration and response to neoadjuvant CRT. We used the median TI of our population as a cut-off value to avoid any introduced selection bias based on clinical data and to provide an agnostic way of categorizing patients solely based on flow cytometry data, regardless of tumor stage and disease biology. Thus, two groups were equal populations of 53 patients with a low and high tumor index, based on the median.

Several factors have been studied regarding the tumor response in neoadjuvant treatment, especially in rectal tumors, including clinical, protein-based, and molecular. The ability to predict TRG is very important for choosing the proper treatment for every individual [27]. To our knowledge, this is the first study to report the role of DNA and tumor index in the evaluation of TRG following neoadjuvant CRT and, additionally, the prognostic role of TI in CRC patient survival.

CD26/DPP4 is a transmembrane glycoprotein that is expressed in many tissue cells but mainly in epithelial cells and lymphocytes. CD26 can act as a tumor suppressor or activator depending on the tumor microenvironment [10]. A few studies indicated that the expression of CD26 was positively associated with CRC tumor stage, degree of differentiation, and development of metastasis, and they highlighted its role as a predictor of poor outcome following curative resection [9,10]. Another recent study showed that CRC patients with diabetes mellitus treated with DPP4 inhibitors showed significantly better 5-year disease-free survival compared to metformin-treated counterparts [28]. These data could emphasize the role of CD26 (+) CRC as a novel prognostic biomarker and as an indicator towards a more individualized treatment. In our study, we examined the presence of CD26 (+) tumor cells in 45 patients. Contrary to the previous studies, we did not confirm any positive association of CD26 expression with advanced CRC stage, tumor differentiation, metastasis, or recurrence potential and survival. Remarkably, there was a negative statistically significant correlation to CRC stage and differentiation (*p*: 0.05). The small sample size (45 patients) might have played a significant role in the presented results, although there was good representation of every CRC stage in this cohort.

One of the key advantages of flow cytometry (FC) in the clinical management of CRC is its ability to provide rapid and reliable intraoperative results. FC allows for the immediate analysis of tumor cell characteristics, including DNA index and aneuploidy, which can inform surgical and therapeutic decision-making rapidly. For instance, the DNA index can help to evaluate tumor responsiveness to neoadjuvant chemoradiotherapy (CRT), enabling oncologists to optimize treatment plans for individual patients. Additionally, fast intraoperative results can assist in identifying patients at higher risk of recurrence, thereby facilitating more aggressive and targeted postoperative interventions. The integration of FC into routine surgical practice holds the potential to significantly enhance the precision of CRC treatment and improve patient outcomes [16].

Based on our results, FC might provide new prognostic and predictive markers in CRC. The major limitation of our study is the small size of our study group. This applies mostly to the role of CD26 expression in CRC cells, which in our study showed divergence from previous publications. More studies are needed to confirm our results and for these markers to be clinically applied. In the future, it would be interesting to investigate the role of the TI and DNA indexes in predicting rectal cancer’s response to neoadjuvant CRT prior to its administration. If our results are also confirmed in the pretreatment state, those indexes could be used for better patient selection for nCRT.

In this study, we have shown another potential of FC to predict the TRG after nCRT and the survival (OS and DFS) of resectable CRC patients. Our results highlight the potential of flow cytometry as a valuable tool in prognosis and treatment planning for colorectal cancer patients. The assessment of DNA index and tumor index through FC offers insights into tumor biology, including aneuploidy and its implications for patient prognosis and response to therapy.

## Figures and Tables

**Table 1:** Study group data analysis regarding tumor characteristics, response to nCRT, tumor recurrence/metastasis, and patient survival.

	Patients (*n*)	Ca Recurrence/Metastasis (*n*)	Alive (*n*)	Ca-Related Death (*n*)	Overall Survival (%)	DFS (%)
Sex						
Male	69	18	51	10	74	62
Female	37	4	30	3	81	78
Tumor location						
Right colon	37	10	28	6	75	65
Left colon	31	7	22	6	71	68
Rectum	36	5	29	1	80	69
Tumor stage						
0	5	0	5	0	100	100
I	20	1	14	1	70	70
II	36	4	32	1	89	80
III	38	13	27	7	71	55
IV	7	4	3	4	43	43
Neoadjuvant therapy	18	2	16	1	89	83
TRG 0	3	0	3	0	100	100
TRG 1	3	0	2	0	67	67
TRG 2	9	1	9	0	100	89
TRG 3	3	1	2	1	67	67
Overall	106	22	81	17	76	72

## Data Availability

The data published in this study are kept confidential by the authors, but part of them could be available, if required.

## Supplementary Materials

The following supporting information can be downloaded at: https://www.mdpi.com/article/10.3390/jcm13164753/s1, Table S1: Study sub-group of patients analysed for CD26 expression. Data analysis regarding tumor characteristics, response to nCRT.
