# Supplementary material for: Could Flow Cytometry Provide New Prognostic Markers in Colorectal Cancer?
Source: J Clin Med. 2024 Aug 13;13(16):4753. doi: 10.3390/jcm13164753 (PMC11355755; doi:10.3390/jcm13164753)
Supplement: Supplementary file 1 [file jcm-13-04753-s001.zip › jcm-3106393-supplementary.pdf]

Table S1. Study sub-group of patients analysed for CD26 expression. Data analysis regarding tumor characteristics, response to nCRT.

CD26 patients	No patients
**Sex**	
Male	29
Female	16
**Tumor location**	
Right colon	16
Left colon	16
Rectum	13
**Tumor stage**	
0	0
I	10
II	16
III	16
IV	3
**Tumor Differentiation**	
Well	1
Moderate	35
Poor	9
**Neoadjuvant therapy**	5
**TRG 0**	0
**TRG 1**	0
**TRG 2**	2
**TRG 3**	3
**Overall**	45
